# A Syndecan-4 Hair Trigger Initiates Wound Healing through Caveolin- and RhoG-Regulated Integrin Endocytosis

**DOI:** 10.1016/j.devcel.2011.08.007

**Published:** 2011-10-18

**Authors:** Mark D. Bass, Rosalind C. Williamson, Robert D. Nunan, Jonathan D. Humphries, Adam Byron, Mark R. Morgan, Paul Martin, Martin J. Humphries

**Affiliations:** 1 School of Biochemistry, University of Bristol, University Walk, Bristol BS8 1TD, UK; 2 Wellcome Trust Centre for Cell-Matrix Research, Faculty of Life Sciences, University of Manchester, Michael Smith Building, Oxford Road, Manchester M13 9PT, UK

## Abstract

Cell migration during wound healing requires adhesion receptor turnover to enable the formation and disassembly of cell-extracellular matrix contacts. Although recent advances have improved our understanding of integrin trafficking pathways, it is not known how extracellular ligand engagement controls receptor dynamics. Using atomic force microscopy, we have measured cell avidity for fibronectin and defined a mechanism for the outside-in regulation of α_5_β_1_-integrin. Surprisingly, adhesive strength was attenuated by the syndecan-4-binding domain of fibronectin due to a rapid triggering of α_5_β_1_-integrin endocytosis. Association of syndecan-4 with PKCα was found to trigger RhoG activation and subsequent dynamin- and caveolin-dependent integrin uptake. Like disruption of syndecan-4 or caveolin, gene disruption of RhoG in mice was found to retard closure of dermal wounds due to a migration defect of the fibroblasts and keratinocytes of RhoG null mice. Thus, this syndecan-4-regulated integrin endocytic pathway appears to play a key role in tissue repair.

## Graphical abstract

**Graphical Abstract:**

**Figure:**
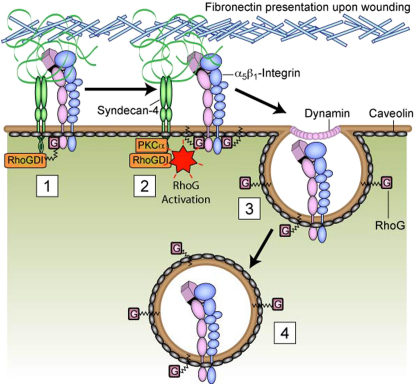

**Highlights:**

► Syndecan-4 engagement mobilizes cells by reducing avidity for extracellular matrix ► Syndecan-4 induces caveolin-mediated endocytosis rather than inactivation of integrin ► Matrix ligands of syndecan-4 cause RhoG activation, which triggers endocytosis ► Gene disruption of syndecan-4, RhoG, or caveolin-1 results in compromised wound healing

## Introduction

The uninjured dermis of adult mammals comprises predominantly collagen and nonmigratory fibroblasts, and contains only very low levels of fibronectin. Upon wounding, blood plasma floods from ruptured vessels into the damaged tissue, triggering recruitment of fibroblasts into the wound bed and leading to wound contraction (Shaw and Martin, 2009). The development of strategies to accelerate or control repair processes requires an understanding of how changes in the extracellular matrix (ECM) are communicated across the plasma membrane to direct cell behavior. α_5_β_1_-integrin is the prototypic fibronectin receptor, but fibronectin-dependent signaling depends upon simultaneous engagement of the transmembrane proteoglycan, syndecan-4, which acts as a fibronectin coreceptor (Morgan et al., 2007). Disruption of the *Sdc4* gene in mice results in delayed wound healing due to compromised motility of fibroblasts, demonstrating a clear role for syndecan-4 in the healing process (Echtermeyer et al., 2001). In vitro, cooperative engagement of syndecan-4 and α_5_β_1_-integrin by fibronectin causes transient activation of Rac1 (Bass et al., 2007), rapid inhibition of RhoA (Bass et al., 2008), and subsequent reactivation of RhoA (Dovas et al., 2006), thus promoting cycles of membrane protrusion followed by cytoskeletal contraction that are necessary for cell migration.

The direct relationship between syndecan-4 and α_5_β_1_-integrin has not been explored. Syndecan-4 activates PKCα by direct association (Koo et al., 2006), and there is evidence that PKCα can cause the activation (Han et al., 2006) or endocytosis of integrin (Ng et al., 1999). Such reports raise the possibility that syndecan-4-dependent PKCα regulation might influence the activity or surface availability of integrins in fibronectin-stimulated fibroblasts. In this study, we use atomic force microscopy (AFM) to measure directly the effect of syndecan-4 engagement on cell avidity for the integrin-binding domain of fibronectin. We find that, contrary to expectation, engagement of syndecan-4 reduces cell avidity for fibronectin by inducing integrin endocytosis. We delineate the signaling pathway by which syndecan-4 induces integrin redeployment, and finally demonstrate that genetic disruption of the key signaling intermediary results in a fibroblast-mediated healing defect. Based on these observations, we redefine the function of syndecan-4 as follows: first, syndecan-4 acts as an integrin regulator, rather than a coreceptor; second, syndecan-4 induces integrin trafficking rather than stabilization; and third, each of these syndecan-4-regulated steps is necessary for efficient wound repair. These advances provide an explanation for how fibroblasts recognize the change in ECM composition following injury and why they become more motile in a fibronectin-rich environment.

## Results

### Engagement of Syndecan-4 Reduces Cell Avidity for Integrin Ligands

**Figure 1:**
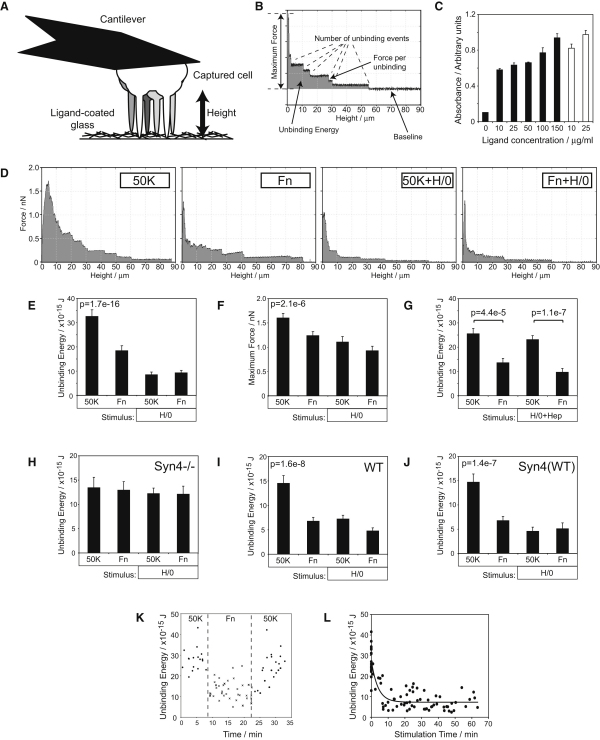
Engagement of Syndecan-4 Reduces Cell Avidity for Fibronectin (A) Individual, cantilever-mounted fibroblasts were contacted with fibronectin fragment-coated surfaces and then retracted. (B) During retraction, deflection of the calibrated cantilever was recorded to obtain measurements of the maximum applicable force, number of individual tethers, and the total work required to detach the cell from the substrate. (C) ELISA analysis of coated ligands to ensure equivalent density of integrin-binding motifs between the integrin-binding fragment of fibronectin (50K, closed bars) and fibronectin (open bars). (D) Unbinding force curves as a fibroblast was withdrawn from 50K or fibronectin in the absence or presence of a soluble syndecan-4-binding fragment of fibronectin (H/0). (E and F) Quantification of the energy (E) or maximum force (F) required to detach a fibroblast. (G) H/0, preincubated with 10 μg/ml heparin, was used as an inactivated syndecan-4 ligand. (H–J) The specific role of syndecan-4 was tested by comparing responses of syndecan-4 null MEFs (H), wild-type MEFs (I), and null MEFs reexpressing the syndecan-4 cDNA (J). (K) Individual unbinding energy measurements as fibroblasts were contacted sequentially with 50K (circles), fibronectin (crosses), and then 50K again. Graph depicts pooled data from three separate cells. (L) Time course of unbinding energy from 50K upon addition of soluble H/0. All values represent at least 80 measurements per condition from five to nine experiments. Error bars represent standard error of the mean; significance was tested by ANOVA. See also Figure S1.

Cell migration requires modulation of the adhesive strength by formation and disassembly of integrin-mediated adhesive contacts. To probe the contributions of integrins and syndecans to the generation of adhesion strength, we used AFM to measure directly the energy required to disengage cells from the ECM. A series of 5 s contacts were made between single fibroblasts, captured on a cantilever, and matrix protein-coated glass (Figure 1A), and the deflection of the cantilever was recorded as the cells were withdrawn from the glass surface (Figure 1B). This arrangement allowed us to record the unbinding energy for the same cell after contact with different surfaces, and thereby enabled avidity changes to be tracked in single cells rather than being averaged across populations. Analysis of individual cells also ensured that all data were obtained from live cells because cell death caused immediate loss of unbinding “steps” and a fall in maximum force. The 5 s contact times were sufficient for integrin binding, but not cytoskeletal reorganization (Taubenberger et al., 2007), allowing specific interrogation of integrin regulation.

To test the effect of syndecan-4 engagement on cell avidity for α_5_β_1_-integrin ligand, cells were contacted with patches of plasma fibronectin, which contains α_5_β_1_-integrin- and syndecan-4-binding motifs, or patches of a recombinant 50 kDa fragment of fibronectin (50K) encompassing the binding site for α_5_β_1_-integrin alone. Equivalent density of integrin ligand on the fibronectin and 50K surfaces was tested and confirmed by ELISA using a monoclonal antibody directed against the integrin-binding domain of fibronectin (Figure 1C).

Withdrawal of a fibroblast from either 50K or fibronectin resulted in an unbinding curve with several features: (1) maximum deflection represented the maximum force that could be applied before integrin tethers were broken; (2) area under the curve represented total energy required to detach a cell from the ECM; and (3) breakage of individual tethers was represented by a series of steps on the curve (Figures 1B and 1D). Because syndecan-4 engagement is known to activate adhesive signals and drive focal adhesion formation (Bass et al., 2007), we had predicted that more energy would be required to detach a cell from fibronectin than 50K. In fact the opposite effect was observed (Figures 1D and 1E). Stimulation of cells with a soluble syndecan-binding fragment of fibronectin, comprising type III repeats 12–15 (H/0), also resulted in low unbinding energy that no longer varied between 50K and fibronectin surfaces, suggesting that the different binding to fibronectin and 50K was due to syndecan engagement (Figures 1D and 1E). A striking feature was the highly reproducible nature of the experiment. Although the absolute values obtained from different cells varied, the same response was observed with every cell tested (see Figure S1A available online). Like unbinding energy, the maximum applicable force also decreased upon syndecan-4 engagement (Figures 1D and 1F).

To establish specific involvement of syndecan-4, we independently blocked either the receptor or ligand. H/0 that had been preincubated with heparin, thus blocking interaction with the syndecan-4 polysaccharide chains, had no effect on unbinding energy (Figure 1G). *Sdc4*^−/−^ MEFs failed to respond to fibronectin or soluble H/0 (Figure 1H), unlike wild-type MEFs or those rescued by re-expression of the full-length syndecan-4 cDNA (Figures 1I and 1J). To identify the integrin regulated by syndecan-4, human fibroblasts were treated with inhibitory monoclonal antibodies. An activity-neutral antibody (K20) had only a very minor effect on unbinding energy, possibly due to steric obstruction, and did not block the response to syndecan-4 engagement by either fibronectin or H/0 (Figure S1B). By contrast, inhibitors of β_1_-integrin (mAb13, Figure S1C) or α_5_-integrin (mAb16, Figure S1D) almost completely blocked binding to 50K or fibronectin. The residual interaction was weaker than that seen after engagement of syndecan-4 and probably due to weak electrostatic interactions of the cell membrane. An inhibitor of α_v_β_3_-integrin (17E6, Figure S1E) had no effect on unbinding energy. These experiments confirm a role for syndecan-4 and demonstrate that the rapid interaction between fibroblasts and fibronectin is mediated by α_5_β_1_-integrin exclusively, rather than α_v_β_3_-integrin, which is noteworthy in itself.

We next used sequential time course analyses to gain further information about the nature of the syndecan-4-integrin relationship. We recorded a series of contacts with 50K, followed by a series of contacts with fibronectin, before making more contacts with 50K. Contact with fibronectin immediately reduced unbinding energy compared to 50K. This very rapid response identifies syndecan-4 as a hair trigger integrin regulator and indicates that receptors and signaling intermediaries are in close proximity even before fibronectin binding (Figures 1K and S1F). Upon returning to 50K, the cell recovered, demonstrating that avidity suppression is a reversible signaling event, and not due to cell damage. Plotting the time course of H/0 action (Figure 1L) confirmed that the reduction in avidity was very fast (t½ = 2.6 min), even though the soluble H/0 was not applied directly to the surface of the cell. The rapid response to diffuse ligand was consistent with the 5 s response when syndecan-4 and integrin were engaged in close proximity by contact with fibronectin. Taken together, these experiments reveal negative regulation of integrin by a molecule previously considered to be a coreceptor.

### Integrin Regulation: Inactivation versus Surface Availability

**Figure 2:**
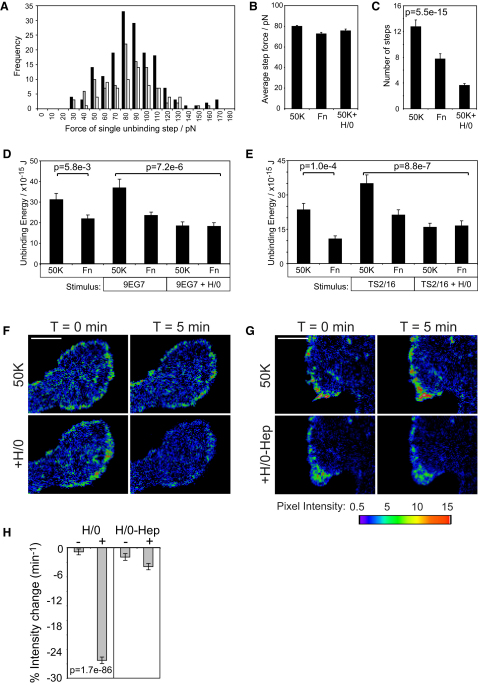
Syndecan-4 Causes Endocytosis Rather than Inactivation of Integrin (A) Frequency distribution of the force required to unbind individual tethers as a cell is retracted from 50K (black), fibronectin (gray), or 50K in the presence of H/0 (white). The graph represents 337 unbinding steps from a single cell. The experiment was repeated on four separate occasions. (B) Average force required during individual unbinding steps. Values represent 1069 unbinding events from four separate experiments. (C) Number of unbinding steps per retraction curve. Values represent the average of 40 curves per condition from 4 separate experiments. (D and E) Response of cells to syndecan-4 engagement after surface integrin is forced into the active conformation using integrin-activating antibodies: 9EG7 (D) or TS2/16 Fab fragment (E). (F–H) Time-lapse TIRF imaging of β_1_-integrin-GFP in the adhesion plane, comparing 5 min prestimulation with 5 min poststimulation when cells were treated with H/0 (F) or H/0 complexed with heparin (G). Scale bars represent 5 μm. (H) Intensity change of 20 adhesions per cell, 9 cells per condition. Error bars represent standard error of the mean; significance was tested by ANOVA. See also Movie S1.

Initial experiments identified reduction in the avidity of cells for fibronectin upon syndecan-4 engagement. The first step in resolving the mechanism was to distinguish whether change in avidity was a consequence of individual bond weakening or a reduction in the number of bonds made. Inspection of the force released at each unbinding event in the original curves revealed similar distribution of step size and similar average step size when cells were detached from 50K, fibronectin, or 50K in the presence of H/0 (Figures 2A and 2B). The consistency in step size indicated that bond strength must be similar under all conditions. Instead, the difference in total force or energy required for detachment was due to a reduction in the number of unbinding steps (Figure 2C). Integrins can be found on the cell surface in active and inactive conformations (Byron et al., 2009), and so the negative effect of syndecan-4 on α_5_β_1_-integrin could be achieved in two ways: reduction in either the activity of integrin or the abundance of integrin on the cell surface. These possibilities were distinguished by forcing α_5_β_1_-integrin into the active conformation using two different monoclonal antibodies (9EG7 and TS2/16; Byron et al., 2009). Application of either antibody increased the energy required to detach cells from 50K (Figures 2D and 2E; p = 0.017) as expected. Despite the enforced activation of surface integrin, engagement of syndecan-4 still caused a reduction in unbinding energy, demonstrating that syndecan-4 engagement lowers avidity by reducing the total number of α_5_β_1_-integrins on the cell surface, rather than by modulating the activation state of individual receptors.

Internalization of β_1_-integrin could be visualized by using total internal reflection fluorescence (TIRF) microscopy to follow disappearance of GFP-β_1_-integrin from the focal plane. Cells spread on 50K adhere using small integrin clusters around the cell periphery. The integrin clusters were stable on 50K, but upon application of H/0 there was a reduction in cluster intensity within 1 min (Figures 2F and 2H and Movie S1A) that did not occur when H/0 was precomplexed with heparin (Figures 2G and 2H and Movie S1B). Thus, both atomic force and time-lapse experiments are supportive of a mechanism involving internalization of β_1_-integrin upon engagement of syndecan-4. In turn, this led us to look for a syndecan-4-dependent mechanism of integrin endocytosis.

### Syndecan-4-Dependent Endocytosis of α_5_β_1_-Integrin

**Figure 3:**
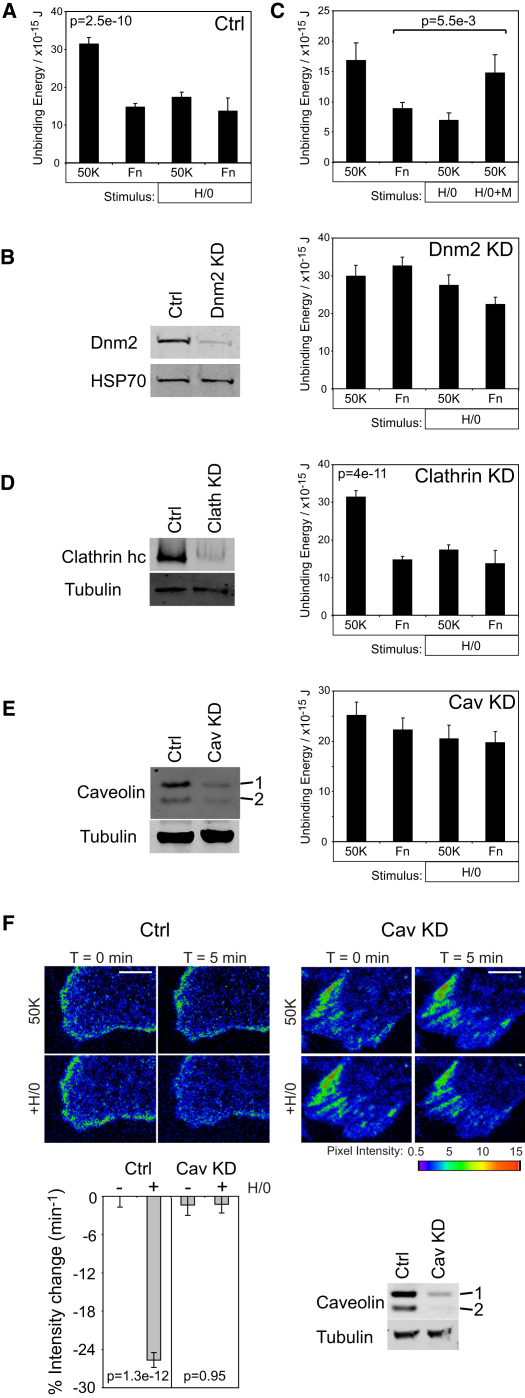
Syndecan-4 Engagement Drives Reduction of Cell Avidity through Dynamin-Dependent, Caveolin-Dependent Endocytosis of α_5_β_1_-Integrin (A, B, D, and E) The effect of syndecan-4 engagement on unbinding energy using fibroblasts transfected with control (Ctrl) (A), dynamin-2-targeted (Dnm2) (B), clathrin heavy-chain-targeted (D), or caveolin-1-targeted (E) siRNA. Western blots compare expression of appropriate molecules between control and siRNA-transfected populations. Knockdown of caveolin-1 caused concomitant loss of caveolin-2, as previously reported (Razani et al., 2001). (C) Unbinding energy of fibroblasts treated with soluble H/0, and subsequently treated with the dynamin inhibitor MiTMAB to block the endocytic signal. (F) Time-lapse TIRF imaging of β_1_-integrin-GFP in the adhesion plane, comparing MEFs transfected with control or caveolin-targeted RNAi oligos. Images represent 5 min pre- or post-H/0 stimulation. Scale bars represent 5 μm. Values represent at least 25 measurements per condition from 4 separate experiments. TIRF analysis summarizes intensity changes of 20 adhesions per cell, 9 cells per condition. Error bars represent standard error of the mean; significance was tested by ANOVA. See also Figure S2 and Movie S2.

Dynamin is responsible for scission of endocytic vesicles from the plasma membrane and integrin internalization (Ng et al., 1999). We used siRNA targeted against the nonneuronal dynamin isoform, dynamin-2, to test the necessity of endocytosis for reduction of unbinding energy. The response of fibroblasts to syndecan-4 engagement was unaffected by transfection with nontargeted siRNA (Figure 3A). However, reduction of dynamin-2 expression to less than 10% of control blocked the reduction in unbinding energy, indicating that syndecan-4 does indeed reduce cell avidity for fibronectin by triggering endocytosis of α_5_β_1_-integrin (Figures 3B and S2A). Furthermore, addition of a pharmacological dynamin inhibitor, MiTMAB, blocked the sustained syndecan-4-induced endocytosis of cells already treated with H/0 and restored unbinding energy to the level observed with 50K alone (Figure 3C). The recovery of unbinding energy indicates that the integrins are in dynamic equilibrium between vesicles and the plasma membrane, and that syndecan-4 engagement simply shifts the equilibrium away from the membrane by increasing endocytosis. α_5_β_1_-integrin is recycled through an Arf6-dependent pathway (Caswell and Norman, 2006). When Arf6 expression was reduced by RNAi, unbinding energy still decreased when a cell was moved from 50K to fibronectin but did not recover upon return to 50K (Figures S2C–S2E). Thus, it appears that active recycling plays a role in integrin redeployment, but the immediate reduction in cell avidity is entirely an endocytic event.

Endocytic pathways can be divided into clathrin-dependent or caveolin-dependent mechanisms (Caswell and Norman, 2006). RNAi-mediated reduction of clathrin heavy-chain expression, to less than 10%, had no effect on the cell response to syndecan-4 ligand (Figure 3D). By contrast, reduction in caveolin expression to less than 20% blocked the reduction in unbinding energy upon syndecan-4 engagement (Figures 3E and S2B). Likewise, reduction of caveolin expression in MEFs blocked H/0-triggered disappearance of GFP-β_1_-integrin from the TIRF plane (Figure 3F). Through these experiments, we identify dynamin- and caveolin-dependent endocytosis of α_5_β_1_-integrin as a consequence of syndecan-4 engagement.

**Figure 4:**
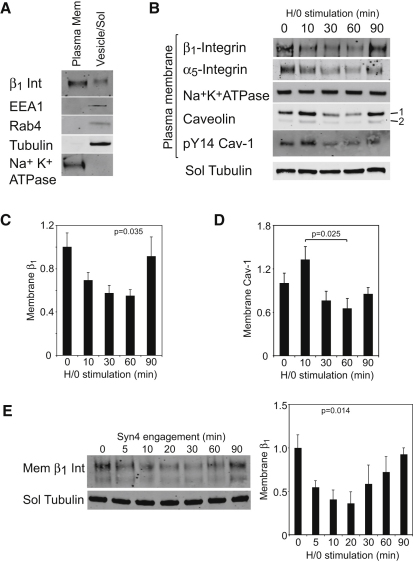
Syndecan-4 Engagement Triggers Biphasic Redistribution of α_5_β_1_-Integrin between the Plasma Membrane and Vesicles (A) Fibroblast membranes were separated by centrifugation into a plasma membrane fraction (Mem) and a small vesicle/soluble (Sol) fraction. Segregation was verified by blotting for vesicle markers (EEA1 and Rab4), soluble marker (tubulin), and plasma membrane marker (Na^+^ K^+^ ATPase transporter). Int, integrin. (B) Western blot analysis of the redistribution of molecules when fibroblasts prespread on 50K were stimulated with H/0 over a time course. (C and D) Quantification of plasma membrane-associated β_1_-integrin (C) and caveolin-1 (Cav-1) (D). (E) Redistribution of plasma membrane-associated β_1_-integrin when fibroblasts in suspension were stimulated with H/0. Error bars represent standard error of the mean of experiments repeated on four to eight separate occasions; significance was tested by ANOVA.

To complement AFM studies, trafficking of integrin and caveolin was investigated further by biochemical fractionation of cells into plasma membrane and vesicle compartments. The segregation of membrane fractions was validated by blotting for marker molecules (Figure 4A). Vesicle markers, early endosome-associated molecule 1 (EEA1) and Rab4, were found exclusively in the small vesicle/soluble fraction, as was tubulin. By contrast, the sodium-potassium ATPase transporter, which is constitutively localized to the cell surface, was restricted to the plasma membrane fraction. As expected, β_1_-integrin was found in both plasma membrane and vesicle fractions (Figure 4A). To test redistribution, fibroblasts were spread onto 50K for 2 hr and then stimulated with H/0 for 0–90 min before fractionating and blotting the plasma membrane fraction. As predicted from AFM, α_5_β_1_-integrin was internalized in response to syndecan-4 engagement, but over a longer time period (90 min) was found to return to the membrane (Figures 4B and 4C). This second phase of redistribution is important because unidirectional integrin internalization would result in cell detachment, whereas a biphasic response would cause integrin redeployment and formation of new adhesion complexes and thereby enable cell migration. The endocytic phase was slower (t½ = 6.0 min) than that detected by atomic force (t½ = 2.6 min) because, in fractionation experiments, integrin is engaged by immobilized 50K that would oppose internalization. Caveolin also redistributed over a similar time period, confirming that syndecan-4 triggers caveolar endocytosis (Figure 4D). Unlike the integrin redistribution, the decrease in membrane-associated caveolin was preceded by a slight increase (Figures 4B and 4D). The same trend was observed for pY14-caveolin (Figure 4B), which is necessary for caveolin uptake (del Pozo et al., 2005). We propose that H/0 triggers immediate internalization of caveolin-coated membrane but that there is also recruitment of membrane-proximal caveolin that enables a large-scale endocytic response.

Each of the experiments thus far has examined the influence of syndecan-4 in the context of simultaneous integrin engagement. If syndecan-4 is to be considered an independent regulator of integrin trafficking in response to fibronectin, one would expect syndecan-4-dependent trafficking of both ligated and ligand-free integrin. H/0 stimulation of fibroblasts in suspension still caused integrin redistribution between plasma membrane and vesicle fractions (t½ = 3.7 min, Figure 4E), demonstrating that syndecan-4 regulates integrin endocytosis, independently of integrin ligation. Collectively, these experiments demonstrate that syndecan-4 is a trigger of caveolar endocytosis that redeploys integrins upon appearance of fibronectin. This model contrasts with the canonical role of syndecan-4 in stabilizing focal adhesions and provides a more logical explanation of how syndecan-4 promotes cell migration during tissue repair.

### Syndecan-4 Drives Endocytosis through the Activation of PKCα and RhoG

**Figure 5:**
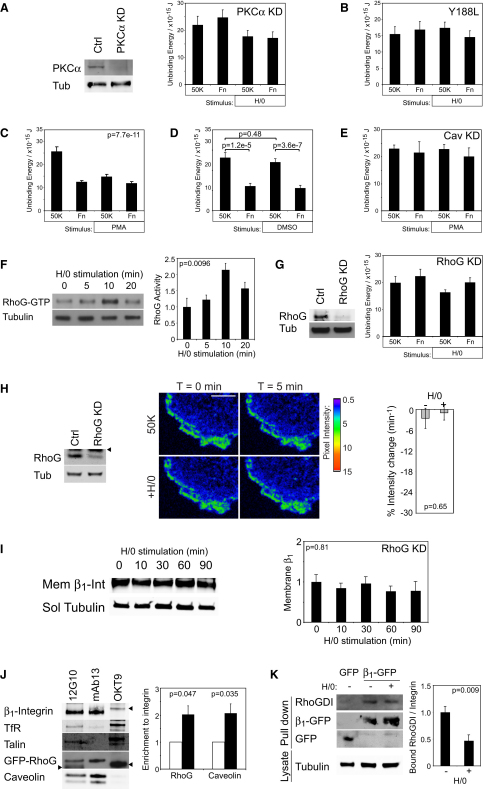
Regulation of PKCα and RhoG Is Responsible for Caveolin-Dependent Endocytosis in Response to Syndecan-4 Engagement (A and B) The effect of syndecan-4 engagement on unbinding energy using MEFs transfected with PKCα-targeted siRNA (A) or expressing a PKCα-binding mutant of syndecan-4 (B). The sequence of signals was resolved by direct activation of PKCα with 100 nM PMA. (C–E) PMA treatment (C), control vehicle solvent (D), and PMA treatment (E) of fibroblasts transfected with caveolin (Cav)-targeted siRNA. (F) Time course of H/0-stimulated activation of RhoG by effector pull-down assay, followed by quantitative western blotting. Gels represent six separate experiments. (G–I) The effect of syndecan-4 engagement on unbinding energy (G), removal of β_1_-integrin-GFP from the TIRF plane (H), and association of β_1_-integrin with the plasma membrane (I), following reduction of RhoG expression by RNAi. Scale bars represent 5 μm. All atomic force measurements represent at least 20 measurements per condition, obtained on 3 separate occasions. (J) Multimolecular complexes associated with active (12G10, white bar) or inactive (mAb13, black bar) β_1_-integrin or transferrin receptor (OKT9) were isolated from fibroblasts expressing GFP-RhoG; recruitment of GFP-RhoG and caveolin relative to β_1_-integrin was determined by quantitative western blotting. Blots were reprobed for talin as a control for selective recruitment to active integrin. (K) H/0-regulated multimolecular complexes associated with β_1_-integrin-GFP were isolated by GFP-Trap from MEFs spread on 50K. Atomic force measurements represent at least 25 measurements per condition from at least 3 separate experiments. Gels represent five separate experiments. Error bars represent standard error of the mean; significance was tested by ANOVA. Arrowheads mark immunoglobulin bands. Ctrl, control; Tub, tubulin. See also Figure S3 and Movie S2.

The redistribution of caveolin, in response to H/0, positions caveolin-dependent endocytosis downstream of syndecan-4, but upstream of α_5_β_1_-integrin, in a signaling cascade. Our next aim was to elucidate the link between syndecan-4 and caveolar trafficking. The cells used in this study express syndecan-1, -2, and -4 (Bass et al., 2007), yet disruption of syndecan-4 expression was sufficient to block fibronectin-dependent endocytosis (Figure 1H). Syndecan-4 is unique among syndecan family members in binding and activating PKCα (Koo et al., 2006), making PKCα a prime candidate to connect syndecan and caveolin. Reduction of PKCα expression to less than 10% by RNAi blocked the reduction of unbinding energy upon syndecan-4 engagement (Figure 5A). Similarly, expression of a PKCα-binding mutant of syndecan-4 (Y188L) failed to rescue the endocytic response in *Sdc4*^−/−^ MEFs (Figure 5B), whereas re-expression of wild-type syndecan-4 restored modulation of unbinding energy (Figure 1J). The effect of H/0 could be mimicked by activating PKC directly with 100 nM phorbol 12-myristate 13-acetate (PMA) (Figure 5C). Treatment of cells with vehicle DMSO had no effect on unbinding energy or the response to fibronectin (Figure 5D). Curiously, the response to PMA was slower than the response to H/0 (H/0, t½ = 2.6 min; PMA, t½ = 7.7 min). We suggest two equally plausible explanations because, first, syndecan-4 is a better activator of PKCα than are lipid activators as suggested previously (Oh et al., 1998), and second, proximity of syndecan-4 to α_5_β_1_-integrin means that syndecan-4 activates PKCα in the correct location, rather than holistically throughout the cell. Cells transfected with the siRNA targeted against caveolin failed to respond to PMA (Figure 5E), placing PKCα between syndecan-4 and caveolin on the signaling cascade. Collectively, these experiments show that the interaction between syndecan-4 and PKCα is necessary for caveolin-dependent endocytosis of integrins, and that the molecules lie in a linear pathway: syndecan-4-PKCα-caveolin-endocytosis.

Our next aim was to identify the pathway activated by PKCα upon syndecan-4 engagement. Although tyrosine phosphorylation of caveolin is necessary for endocytosis (Cao et al., 2002), there are no reports that serine/threonine phosphorylation of caveolin by PKCα is important. One possible endocytic mechanism stems from the recent identification of a complex comprising syndecan-4, RhoGDI, and RhoG (Elfenbein et al., 2009). Elfenbein et al. (2009) reported a syndecan-4-dependent phosphorylation of RhoGDI in response to growth factor stimulation, which led to release and activation of RhoG. RhoG has been linked to two processes: activation of Rac1 through the ELMO/Dock180 complex (Katoh et al., 2006) and caveolar endocytosis of growth factor receptors (Samson et al., 2010). Wild-type RhoG traffics from the plasma membrane to caveolar vesicles, whereas constitutively active RhoG accumulates preferentially in vesicles and accelerates internalization of membrane cargoes (Prieto-Sánchez et al., 2006). We hypothesized that RhoG might also trigger integrin endocytosis upon engagement of syndecan-4 by fibronectin. We tested fibronectin-dependent activation of RhoG by effector pull-down using the N terminus of ELMO2 as bait. H/0 stimulation of fibroblasts spread on 50K caused a rapid increase in RhoG activity within 10 min (Figure 5F), correlating with internalization of β_1_-integrin (Figure 4C). Reduction of RhoG expression to less than 10% of control by RNAi blocked the syndecan-4-dependent reduction in unbinding energy (Figures 5G and S3), disappearance of GFP-β_1_-integrin from the TIRF plane (Figure 5H and Movie S2), and loss of β_1_-integrin from the plasma membrane fraction (Figure 5I), demonstrating that syndecan-4-dependent activation of RhoG is indeed the trigger for integrin endocytosis.

We have already conjectured that the 5 s response to fibronectin (Figure 1K) necessitates the existence of a preformed endocytic complex, and so we immunoprecipitated β_1_-integrin from cells expressing GFP-RhoG. Not only did RhoG precipitate with β_1_-integrin, but it was enriched in the complex associated with inactive integrin (mAb13, Figure 5J). Caveolin was also enriched in the inactive integrin complex, indicating that ligand-free integrin is primed for redeployment through an endocytic pathway. As expected, talin was recruited preferentially to active integrin (12G10). Neither RhoG nor caveolin was recruited to the control transferrin receptor complex (OKT9). Preferential redeployment of inactive β_1_-integrin, rather than ligated integrin, refines the model of fibronectin-induced integrin mobilization and explains why syndecan-4 induces integrin redeployment, rather than cell detachment. The second necessary feature of the integrin-RhoG complex is that RhoGDI should be present in a preformed complex and be released upon syndecan-4 engagement. RhoGDI was indeed detected in the complex when total GFP-β_1_-integrin, but not GFP alone, was immunoprecipitated from MEFs spread on 50K, using a GFP-Trap (Figure 5K). Ten minute stimulation with H/0, which coincided with the peak in RhoG activity, caused a 46% reduction in bound RhoGDI. Together, these experiments demonstrate that engagement of syndecan-4 by fibronectin causes release of RhoGDI from a β_1_-integrin complex and triggers a wave of RhoG activity that preferentially redeploys inactive α_5_β_1_-integrin through an endocytic mechanism. Combined with the existing knowledge of the links from syndecan-4 to RhoG through PKCα and RhoGDI, we have delineated the molecular pathway responsible for integrin internalization upon exposure to fibronectin.

### The Syndecan-4-RhoG-Caveolin Endocytic Cascade Is Necessary for Wound Closure

**Figure 6:**
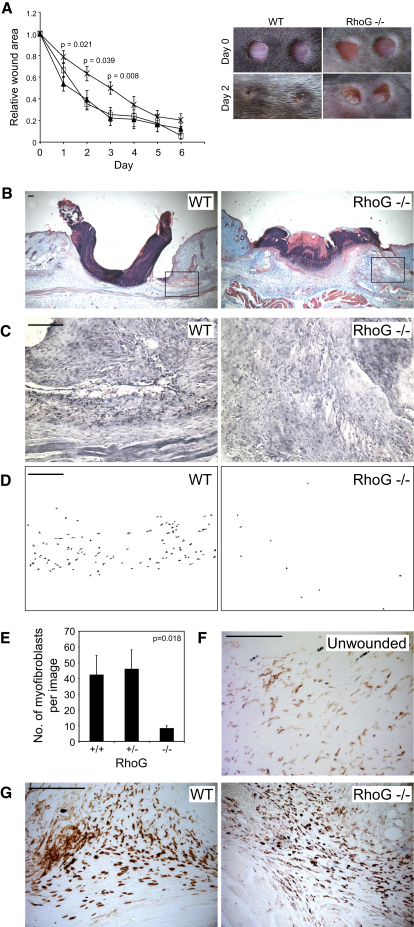
RhoG Expression Is Required for Efficient Wound Closure (A) Closure of 4 mm punch wounds in 7-week-old mice was compared between *Rhog*^−/−^ mice (cross), wild-type (closed triangle), and heterozygous (open square) littermates. (B) Trichrome staining of skin sections to show comparable tissue morphology but compromised wound contraction of *Rhog*^−/−^ mice; squares indicate the regions analyzed for myofibroblast recruitment. (C–G) Myofibroblast recruitment was measured by α-smooth muscle actin staining (C), generation of intensity threshold masks (D), and automated quantification of positive cells (per 0.23 mm^2^ image) (E) from wild-type and *Rhog*^−/−^ mice. Density of macrophages was compared between unwounded (F) and wounded skin (G) of wild-type and *Rhog*^−/−^ mice. Data represent 14 wounds per genotype. Error bars represent standard error of the mean; significance was tested by ANOVA. Scale bars represent 100 μm.

Having identified RhoG as a key molecular link between syndecan-4 and caveolin-dependent endocytosis in vitro, we investigated the role of this pathway in vivo. Both *Sdc4*^−/−^ and *Cav1*^−/−^ mice have similar and surprisingly mild phenotypes. Both are viable and fertile, and suffer a delay in wound closure that has been attributed to a defect in fibroblast migration (Echtermeyer et al., 2001; Grande-García et al., 2007; Razani et al., 2001). *Cav1*^−/−^ mice also suffer nondetrimental hyperproliferation of some cell types. We hypothesized that if the healing defects of the two mice are a consequence of a disruption in the syndecan-4-RhoG-caveolin signaling cascade, then *Rhog*^−/−^ mice would suffer a similar defect. The *Rhog*^−/−^ mouse is also viable and fertile, and the only documented phenotype is a slightly enhanced immune response (Vigorito et al., 2004). We investigated the closure of 4 mm punch wounds in 7-week-old *Rhog*^−/−^ mice by imaging the live mice for 6 days postwounding, and measuring the wound area digitally. Compared to wild-type or heterozygous littermates, *Rhog*^−/−^ mice exhibited a striking delay in wound closure, with a significantly larger wound area at 1–3 days (Figure 6A). By day 5, the wound areas of wild-type, heterozygous, and knockout mice were comparable. Defects in wound contraction are generally a consequence of impaired fibroblast migration into the defect, where some subsequently differentiate into contractile myofibroblasts (Shaw and Martin, 2009). Macroscopic or histological inspection of the *Rhog*^−/−^ mice suggested such a defect (Figures 6A and 6B). Thus, histology of 3 day skin sections revealed the appearance of α-smooth muscle actin-positive myofibroblasts around the wound sites of wild-type and heterozygous mice. In *Rhog*^−/−^ mice, the number of α-smooth muscle actin-positive cells was reduced to 20%, and positive cells were scattered, indicating a migration defect in the precursor fibroblasts (Figures 6C–6E). Other cell migration events at the wound site induced by wounding include the recruitment of leukocytes to chemoattractants released by the wounded epithelium and degranulating platelets. Compared to unwounded skin (Figure 6F), F4/80-positive macrophages were recruited in similar numbers to the wounds of both wild-type and *Rhog*^−/−^ mice (Figure 6G). The difference between fibroblast and macrophage recruitment suggests that RhoG is necessary for migration toward syndecan-4 ligands, such as fibronectin, but not other chemoattractants. Thus, *Rhog*^−/−^ mice exhibited a delay in wound contraction that resembled the reported defect of *Sdc4*^−/−^ mice (Echtermeyer et al., 2001) and was indicative of a migration defect of dermal fibroblasts, but not immune cells.

**Figure 7:**
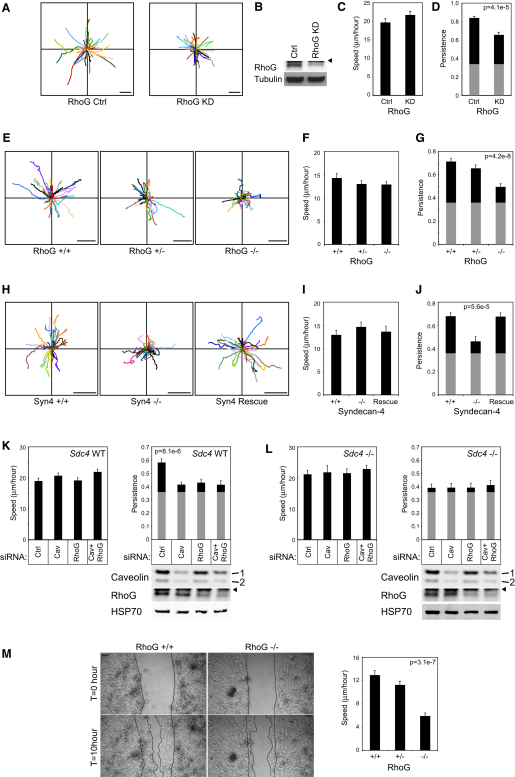
Migration of RhoG or Syndecan-4-Defient Fibroblasts over a Fibrillar Matrix (A–L) Migration paths (A, E, and H), average speed (C, F, I, K, and L), and average persistence (= displacement/total distance moved) (D, G, and J–L) of cells migrating over a cell-derived matrix. Gray boxes indicate the minimum possible persistence values when cells migrate randomly on homogeneous matrix. Cells tracked were as follows: immortalized human fibroblasts transfected with control or RhoG-targeted siRNA (A–D), including analysis of expression of RhoG by western blot (B); primary E13.5 MEFs from *Rhog*^−/−^ mice, wild-type, and heterozygous littermates (E–G); immortalized MEFs from wild-type and *Sdc4*^−/−^ littermates and MEFs rescued by endogenous syndecan-4 expression (H–J); and wild-type (K) and *Sdc4*^−/−^ (L) MEFs following transfection with caveolin- or RhoG-targeted siRNA. Data represent analysis of over 100 cells per condition, from 3 separate experiments. (M) Ten hour scratch assay of primary keratinocytes isolated from neonatal *Rhog*^−/−^ mice, wild-type, and heterozygous littermates. Error bars represent standard error of the mean; significance was tested by Kruskal-Wallis tests for nonparametric data. Scale bar represents 100 μm. Arrowheads mark immunoglobulin bands. See also Figure S4 and Movies S3 and S4.

Fibroblast migration along linear fibronectin fibers can be modeled by tracking migration over a fibrillar cell-derived matrix, which reflects the wound environment in vivo (Figure S4). To test the contribution of RhoG to fibronectin-guided migration, RhoG-deficient cells were generated by either RNAi-mediated reduction of RhoG in human fibroblasts or isolation of primary embryonic fibroblasts from the transgenic mice. In both cases, reduced RhoG expression compromised the ability of fibroblasts to proceed persistently over a fibrillar matrix but had no effect on speed of migration (Figures 7A–7G and Movie S3). The persistence defect resembled that of *Sdc4*^−*/*−^ MEFs (Figures 7H–7J), which fail to detect and respond to fibronectin (Bass et al., 2007). If the signal from syndecan-4 to RhoG to caveolin were linear, as proposed, one would expect disruption of multiple components to have no additional effect on persistence, once the cascade is broken. Therefore, we combined knockdown of caveolin and RhoG in wild-type or *Sdc4*^−/−^ MEFS, and found that removal of multiple molecules had no greater effect than removal of a single component (Figures 7K and 7L). Together, these experiments identify the syndecan-4-RhoG-caveolin pathway as a regulator of directional migration that enables fibroblast immigration as part of a healing response.

Fibroblasts are not the only cell type to exhibit altered syndecan-4 expression upon skin wounding. Keratinocytes upregulate syndecan-4 expression around a wound (Gallo et al., 1996) and, like fibroblasts, exhibit altered adhesion characteristics when exposed to the H/0 fragment of fibronectin (Wilke et al., 1993). We isolated primary keratinocytes and found that *RhoG*^−/−^ keratinocytes exhibited reduced migration when compared with wild-type or heterozygous equivalents in a scratch wound assay (Figure 7M and Movie S4). Therefore, when analyzing cell types that migrate during a wound response, we find that migration of fibronectin-responsive cell types depends on the RhoG signaling axis, whereas others, such as macrophages, do not. When coupled with the fact that syndecan-4 and RhoG knockouts are nonlethal, unlike integrin gene deletions (Hynes, 2002), the role of syndecan-4 in controlling fibronectin-dependent integrin redeployment during the healing process makes syndecan-4 a logical therapeutic target for the manipulation of integrin-dependent migration.

## Discussion

In this study, we have elucidated a signaling pathway that controls the cell-surface delivery of α_5_β_1_-integrin during wound healing. Intriguingly, this pathway is initiated by ligand binding to a distinct adhesion receptor, syndecan-4, which therefore identifies the latter as a primary sensor of the adhesive microenvironment. Our key findings are: (1) syndecan-4 engagement reduces cell avidity for fibronectin, contrary to expectation; (2) syndecan-4 regulates surface availability, rather than activity of α_5_β_1_-integrin; (3) syndecan-4 triggers rapid endocytosis of preformed integrin complexes, enriched in inactive integrin, through a caveolin-dependent pathway; (4) activation of RhoG, downstream of PKCα, mediates the endocytic signal; and (5) *Sdc4*^−/−^, *Rhog*^−/−^, and *Cav1*^−/−^ mice suffer similar wound closure defects, indicating the importance of this signaling cascade in fibroblast migration episodes in vivo. Collectively, these findings describe a model of integrin redeployment whereby exposure of fibroblasts to fibronectin, upon wounding, results in fibroblast migration to repair the defect.

Our observation of the reduction in cell avidity for fibronectin was initially surprising because many studies have described syndecan-4-dependent reinforcement of focal adhesions by recruitment of cytoskeletal proteins such as vinculin (Bass et al., 2007). However, enhanced integrin trafficking would be consistent with syndecan-4-regulated activation of Rac1-dependent membrane protrusion (Bass et al., 2007) and inhibition of RhoA-dependent contraction (Bass et al., 2008) at the leading edge. Activation of RhoG and Rac1 and redeployment of α_5_β_1_-integrin could be classed as early-phase migratory events and define syndecan-4 as the initial sensor for fibronectin, which initiates fibroblast migration into the wound bed. Because cell migration requires cycles of membrane protrusion and cytoskeletal contraction, the stabilization of integrin-containing adhesions behind the leading edge, through the reactivation of RhoA (Dovas et al., 2006), could be classed as late-phase events and will play an equally important role in fibroblast migration.

If we are to understand fibronectin-dependent signaling fully, further investigation will be required to resolve the interdependence of GTPase activation and receptor trafficking. The Rac1 regulation that we reported previously (Bass et al., 2007) does not appear to be a direct consequence of α_5_β_1_-integrin trafficking because H/0 stimulation of fibroblasts in suspension causes integrin redistribution (Figure 5H), but not Rac1 regulation (Bass et al., 2007). Localization of active Rac1 to the leading edge is known to be integrin dependent (del Pozo et al., 2004) and mediated by the sequestration of pY14-caveolin, whereas GTP loading of Rac1 is syndecan-4 dependent (Bass et al., 2007) and may even require clathrin-dependent endocytosis of inactive Rac1 (Palamidessi et al., 2008). Conversely, Rac1 activation does not appear to be the driving force behind integrin endocytosis because active Rac1 does not associate with caveolar vesicles or drive endocytosis in the same way as active RhoG (Prieto-Sánchez et al., 2006) and could not be detected in our integrin immunoprecipitation experiments. Thus, it appears that each of the protrusive signals is interconnected but that none is exclusively dependent on another. The next challenge will be the determination of how integrin organizes the caveolin microdomains, necessary for localized Rac1 signaling, and whether syndecan-4 regulates the process.

It is interesting to speculate that the temporal coordination of events may well depend on the proximity of the molecules involved. The very short response time of cells touched onto fibronectin is consistent with the endocytosis of a preformed complex, and we have demonstrated biochemically the presence of RhoG and caveolin in a β_1_-integrin complex. Syndecan-4 is reported to colocalize with integrins in focal adhesions (Woods et al., 2000), but the distribution of unengaged receptors is not resolved. If we extrapolate the model of hair trigger endocytosis, we would predict the association of α_5_β_1_-integrin and syndecan-4 in microclusters with caveolin and RhoG. The force required to break individual interactions between α_5_β_1_-integrin and fibronectin has been recorded as 39 ± 8 pN (Sun et al., 2005). The force release events that we record during cell detachment range from 37 to 175 pN (Figure 2A); these data are consistent with the presence of microclusters containing one to five α_5_β_1_-integrins. The fact that the force distribution does not change after H/0 stimulation suggests that the mechanism of syndecan-4 action is not based upon increased clustering of unengaged α_5_β_1_-integrin. The more rapid response to H/0 than PKC-activator, PMA action, also relies on proximity of syndecan-4 and α_5_β_1_-integrin, even before fibronectin binding. We speculate that the complete α_5_β_1_-integrin-syndecan-4 cluster is internalized in response to syndecan-4 engagement, and although a paucity of antibodies has prevented direct demonstration of this point, vesicular colocalization of syndecan-2 with β_1_-integrin has been reported (Zimmermann et al., 2005). The generation of improved reagents for detecting syndecan-4, followed by high-resolution analyses of receptor distribution, is a high priority.

The role of syndecan-4 as a sensor of tissue damage in vivo is now apparent, and identification of molecules downstream of syndecan-4 has allowed us to resolve the mechanism of fibroblast recruitment during tissue repair. *Sdc4*, *RhoG*, and *Cav1* null mice each share a wound healing defect that can be attributed to a loss of persistent fibroblast migration (Figures 6 and 7; Echtermeyer et al., 2001; Grande-García et al., 2007). Although the fundamental roles of α_5_β_1_-integrin and Rac1 in development render null mice embryonic lethal, there is a clear role for these molecules in tissue repair. Expression of α_5_β_1_-integrin is induced upon wounding (Hynes, 2002), and mice with a fibroblast-specific Rac1 deletion suffer healing delay, wound contraction, and fibroblast migration defects that are comparable to those observed in the *Sdc4*^−/−^ mouse (Liu et al., 2009). The possibility of syndecan-4 modulating the behavior of integrin and Rac1, without the deleterious side effects associated with targeting the molecules directly, makes syndecan-4 an attractive target for wound treatment.

## Experimental Procedures

### Sample Preparation

For all experiments cells were treated with 25 μg/ml cycloheximide for 2 hr prior to detachment and throughout the experiment, to prevent synthesis of fibronectin and other syndecan-4 ligands.

### AFM

Force measurements were made using a CellHesion200 atomic force head (JPK Instruments). Cells were mounted on 50K-coated tipless silicon SPM-sensor cantilevers (NanoWorld), using Brightfield optics to ensure the capture of individual cells. Captured cells were allowed to rest for 20 min before taking measurements, and in each case the first measurement was discarded. Cantilever-mounted cells were contacted with FluoroDish coated with patches of fibronectin and 50K, at equivalent ligand density, with an applied force of 1 nN, a 5 s contact time, and 45 s intervals between measurements to allow cell recovery. For stimulation experiments, baseline measurements of unbinding from 50K and fibronectin were made before injecting the soluble stimulant into the dish. Force curves were analyzed using JPK data analysis software with baseline and tilt correction. For analysis of force release of individual unbinding events, steps were fitted with a significance of p < 0.001.

### TIRF Microscopy

Internalization of β_1_-integrin-GFP was recorded by TIRF at a 70 nm penetration depth. Images were acquired for 5 min on 50K before addition of H/0 or H/0 complexed with heparin (10 μg/ml each) and image acquisition for a further 5 min. Movies were analyzed, using ImageJ, by outlining 20 focal adhesions per cell and recording depreciation in intensity over 1 min segments, ensuring that the analysis area remained centered over the adhesion for the duration of analysis.

### Membrane Fractionation

Cells spread on 50K-coated dishes and stimulated with 10 μg/ml H/0 were harvested at 4°C by scraping in PBS containing calcium and magnesium. Membranes were fragmented by three 12 J pulses using a Vibra-Cell sonicator (Sonics), before removing nuclear debris with a 10 min, 1000 × g centrifugation step. Membranes were separated into plasma membrane pellet and vesicle/soluble supernatant by 10 min centrifugation at 10,000 × g.

### GTPase Activity Assay

Active RhoG was affinity precipitated using GST-ELMO2 (amino acids 1–362).

### Integrin Complexes

Activity-specific multimolecular integrin complexes were isolated on paramagnetic beads, coated with 200 μg/ml antibody. Fibroblasts expressing GFP-RhoG were incubated with beads for 20 min at room temperature and complexes stabilized with DTBP membrane-permeable crosslinker, before lysing cells by sonication. Matrix-dependent integrin complexes were isolated by GFP-Trap capture of GFP-β_1_-integrin from MEFs spread on 50K-coated dishes, stimulated with H/0, and crosslinked with DTBP.

### Wound Healing

All experiments were carried out at the University of Bristol according to UK Home Office regulations. Seven 7-week-old mice were anesthetized by isoflurane inhalation. Four full-thickness excisional wounds were made on the shaved back on either side of the dorsal midline with a 4 mm biopsy punch (Kai Industries). Wound closure was recorded macroscopically by photographing the live mice daily until the wound had closed, and analyzing images using ImageJ. Histological analysis was conducted 3 days postwounding.

### Migration Analysis

Cell-derived matrices were generated as described previously (Bass et al., 2007) by culturing confluent fibroblasts for 10 days before removing the fibroblasts by NH_4_OH lysis. For migration, cells were spread for 4 hr before imaging every 10 min for 10 hr. The migration paths of all nondividing, nonclustered cells were tracked, and persistence was determined by dividing linear displacement of a cell by the total distance migrated.

## Supplemental Information

Document S1. Four Figures and Supplemental Experimental Procedures

Movie S1. Induction of Integrin Endocytosis by Syndecan-4, Related to Figure 2 MEFs expressing β_1_-integrin-GFP were filmed by TIRF microscopy at two frames per second and displayed in rainbow false-color. A MEF spread on 50K was filmed for 5 min, during which time integrin in the adhesion plain was constant. Addition (white flash) of H/0 (A) induced disappearance of integrin from the adhesion plane over the following 5 min, whereas addition of H/0 precomplexed with heparin (B) did not.

Movie S2. Caveolin and RhoG Expression Are Necessary for Syndecan-4-Dependent Integrin Endocytosis, Related to Figures 3 and 5 MEFs expressing β_1_-integrin-GFP and transfected with appropriate siRNAs were filmed by TIRF microscopy at 0.5 frames per second and displayed in rainbow false-color. MEFs spread on 50K were filmed for 5 min, during which time integrin in the adhesion plain was constant. Upon addition of H/0 (white flash), integrin disappeared from the adhesion plane of MEFs transfected with nontargeting siRNA (A) but remained unaffected in MEFs transfected with an siRNA targeted against caveolin-1 (B) or RhoG (C).

Movie S3. RhoG Expression Is Necessary for Persistent Fibroblast Migration, Related to Figure 7 Human fibroblasts transfected with a nontargeting control siRNA (A) or siRNA targeted against RhoG (B) were tracked migrating over fibrillar cell-derived matrix for 10 hr at six frames per hour.

Movie S4. RhoG Expression Influences Keratinocyte Migration, Related to Figure 7 Confluent monolayers of primary keratinocytes isolated from wild-type (A and C) or *RhoG*^−/−^ (B and D) littermates were scratched and filmed at four frames per hour for 10 hr at low power (A and B) or 6 hr at high power (C and D).
