# A Syndecan-4 Hair Trigger Initiates Wound Healing through Caveolin- and RhoG-Regulated Integrin Endocytosis

**DOI:** 10.1016/j.devcel.2012.10.021

**Published:** 2012-11-13

**Authors:** Mark D. Bass, Rosalind C. Williamson, Robert D. Nunan, Jonathan D. Humphries, Adam Byron, Mark R. Morgan, Paul Martin, Martin J. Humphries

(Developmental Cell *21*, 681–693, October 18, 2011)

It has come to the authors’ attention that during figure composition, a copy of the caveolin blot from Figure 7K was inadvertently overlaid on top of the caveolin blot in Figure 7L. We have deleted this extraneous panel to reveal the correct blot underneath, which was present in the originally accepted version of the figure. The correct version of the figure is printed below.

## Figures and Tables

**Figure 7:**
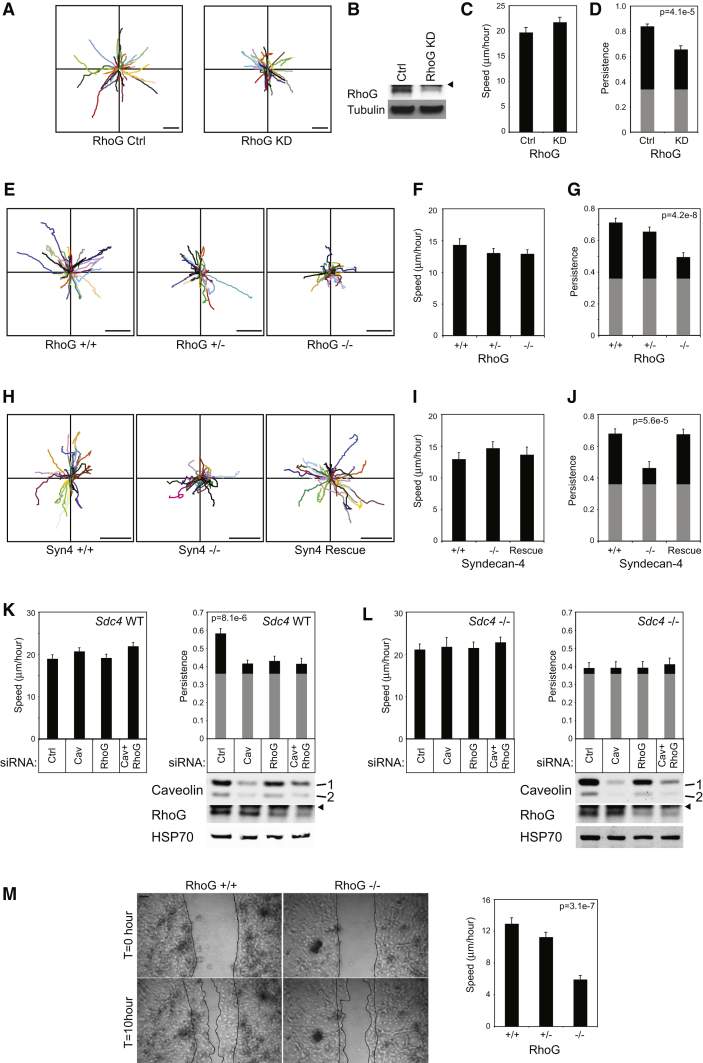
Migration of RhoG or Syndecan-4-Defient Fibroblasts over a Fibrillar Matrix (A–L) Migration paths (A, E, and H), average speed (C, F, I, K, and L), and average persistence ( = displacement/total distance moved) (D, G, and J–L) of cells migrating over a cell-derived matrix. Gray boxes indicate the minimum possible persistence values when cells migrate randomly on homogeneous matrix. Cells tracked were as follows: immortalized human fibroblasts transfected with control or RhoG-targeted siRNA (A–D), including analysis of expression of RhoG by Western blot (B); primary E13.5 MEFs from *Rhog*^−/−^ mice, wild-type, and heterozygous littermates (E–G); immortalized MEFs from wild-type and *Sdc4*^−/−^ littermates and MEFs rescued by endogenous syndecan-4 expression (H–J); and wild-type (K) and *Sdc4*^−/−^ (L) MEFs following transfection with caveolin- or RhoG-targeted siRNA. Data represent analysis of over 100 cells per condition, from 3 separate experiments. (M) Ten hour scratch assay of primary keratinocytes isolated from neonatal *Rhog*^−/−^ mice, wild-type, and heterozygous littermates. Error bars represent standard error of the mean; significance was tested by Kruskal-Wallis tests for nonparametric data. Scale bar represents 100 μm. Arrowheads mark immunoglobulin bands. See also Figure S4 and Movies S3 and S4.

