# Supplementary material for: A Syndecan-4 Hair Trigger Initiates Wound Healing through Caveolin- and RhoG-Regulated Integrin Endocytosis
Source: Dev Cell. 2011 Oct 18;21(4):681–93. doi: 10.1016/j.devcel.2011.08.007 (PMC3202633; doi:10.1016/j.devcel.2011.08.007)
Supplement: Document S1. Four Figures and Supplemental Experimental Procedures [file mmc1.pdf]

Developmental Cell, Volume *21*

Supplemental Information

A Syndecan-4 Hair Trigger Initiates Wound Healing through Caveolin- and RhoG-Regulated Integrin Endocytosis

Mark D. Bass, Rosalind C. Williamson, Robert D. Nunan, Jonathan D. Humphries, Adam Byron, Mark R. Morgan, Paul Martin, and Martin J. Humphries

Supplemental Inventory

Supplemental Figure 1: Reproducibility and integrin specificity of cell attachment to fibronectin, related to Fig. 1.

Supplemental Figure 2: Alternative oligonucleotide sequences targeted against dynamin or caveolin also block syndecan-4-induced endocytosis. Arf6 is not responsible for syndecan-4-regulated reduction in cell avidity, but mediates recycling of $\alpha_5\beta_1$ integrin, related to Fig. 3.

Supplemental Figure 3: An alternative oligonucleotide sequence targeted against RhoG also blocks syndecan-4-induced endocytosis, related to Fig. 5.

Supplemental Figure 4: Cell-derived fibrillar matrix, related to Fig. 7.

Supplemental Movie 1: Induction of integrin endocytosis by syndecan-4, TIRF movie related to Fig. 2.

Supplemental Movie 2: Caveolin and RhoG expression are necessary for syndecan-4-dependent integrin endocytosis, TIRF movie related to Fig. 3+5.

Supplemental Movie 3: RhoG expression is necessary for persistent fibroblast migration over a fibrillar matrix, related to Fig. 7.

Supplemental Movie 4: RhoG expression influences keratinocyte migration, related to Fig. 7.

Extended Experimental Procedures.

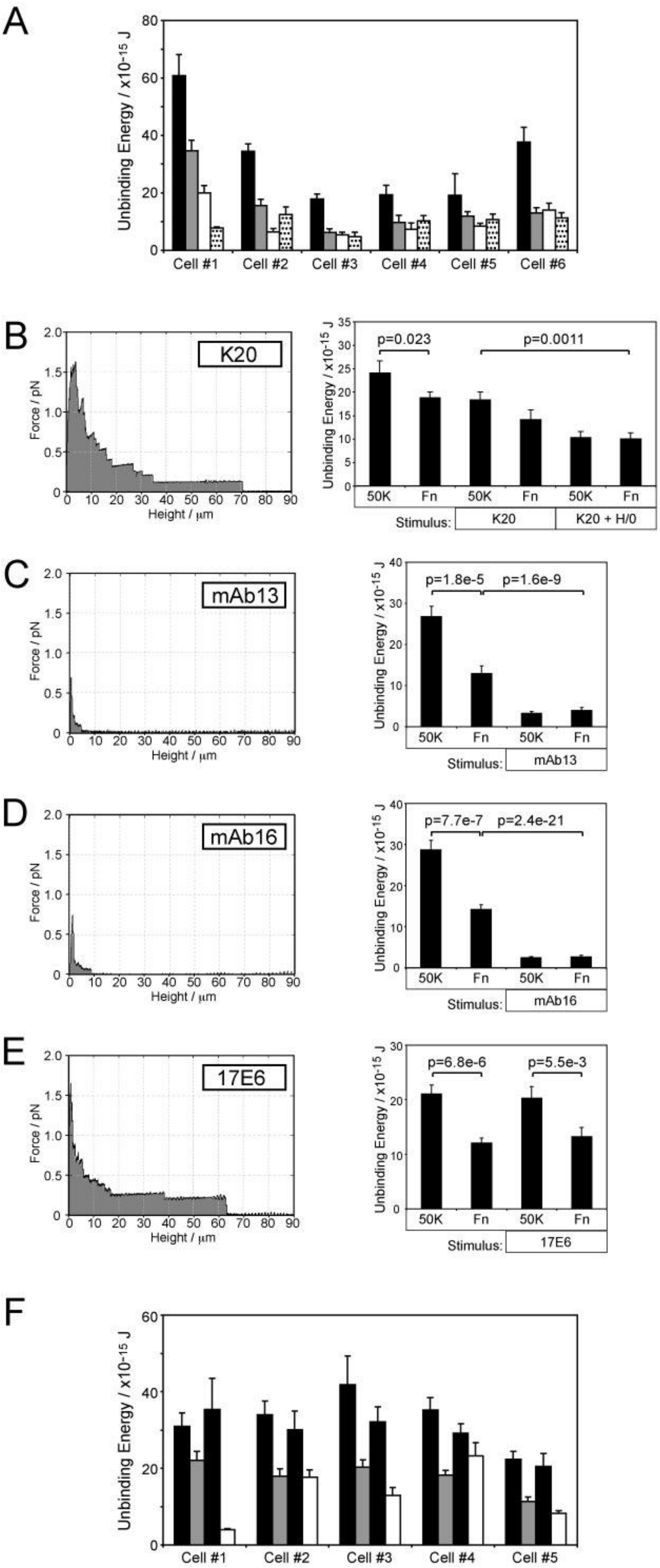
A
Unbinding Energy / x10-15 J
0
20
40
60
80
Cell #1
Cell #2
Cell #3
Cell #4
Cell #5
Cell #6
B
K20
Force / pN
Height / μm
p=0.023
p=0.0011
50K
Fn
Stimulus:
K20
K20 + H/0
C
mAb13
p=1.8e-5
p=1.6e-9
D
mAb16
p=7.7e-7
p=2.4e-21
E
17E6
p=6.8e-6
p=5.5e-3
F

Figure S1. Reproducibility and integrin specificity of cell attachment to fibronectin, related to Fig. 1. (A) Unbinding energies of individual cells touched onto 50K (black), fibronectin (grey), 50K in the presence of soluble H/0 (white) or fibronectin in the presence of H/0 (speckled). (B) Unbinding energies of individual cells sequentially touched onto 50K (black), fibronectin (grey), 50K again (black) or 50K in the presence of soluble H/0 (white). Bars represent 10 measurements per cell, error bars represent standard error. A series of experiments were conducted to identify the receptors involved in the fibronectin response. The following monoclonal antibodies were used to identify the integrin regulated by syndecan-4: K20, a neutral antibody against β_1 integrin (C); mAb13, an inhibitory antibody against β_1 integrin (D); mAb16, an inhibitory antibody against α_5 integrin (E); 17E6, an inhibitory antibody against α_v integrin (F). Values represent averages of at least 25 measurements per condition obtained on 3 separate occasions, error bars represent standard error, significance was tested by ANOVA.

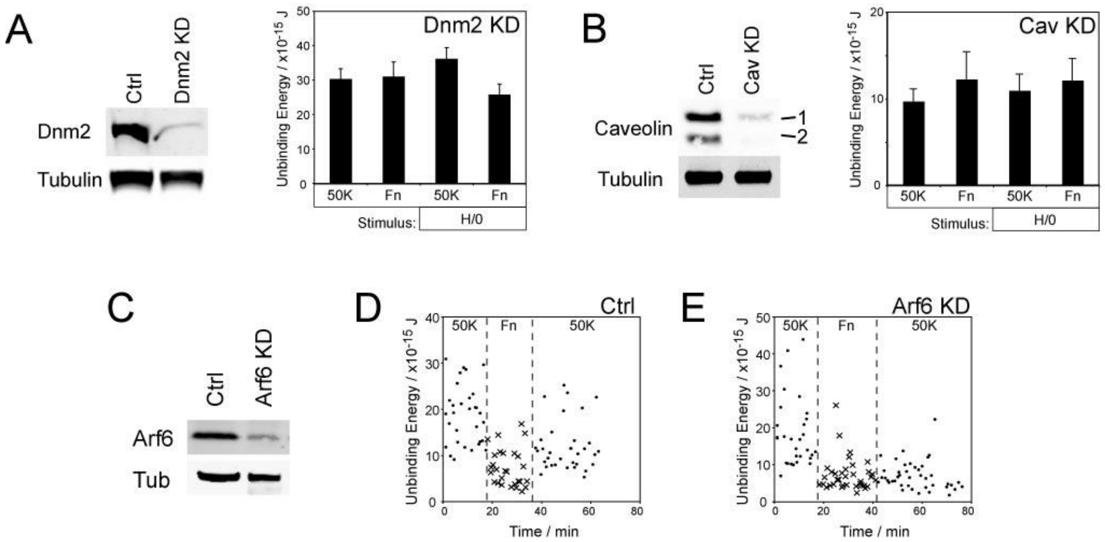

Figure S2. Syndecan-4-driven reduction of cell avidity occurs through dynamin-, caveolin-dependent endocytosis of $\alpha_5\beta_1$ integrin, rather than Arf6-dependent recycling, related to Fig. 3. The effect of syndecan-4 engagement on unbinding energy using fibroblasts transfected with dynamin-2-targeted (A) or caveolin-1-targeted (B) siRNA. These experiment are repeats of those displayed in Fig. 3B and 3E, respectively, using alternative siRNA sequences. Western blots compare expression of appropriate molecules between control and siRNA-transfected populations. (C) Arf6 expression of MEFs transfected with control or Arf6-targeted siRNA. Individual unbinding energy measurements of MEFs transfected with control (D) or Arf6-targeted (E) siRNA. Contacts were made sequentially with 50K (closed circles), fibronectin (crosses), and 50K again. Values represent at least 10 measurements per condition from 3 separate experiments. Error bars represent standard error.

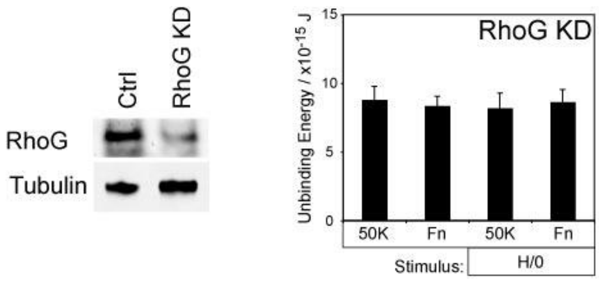

Figure S3. Syndecan-4-driven reduction of cell avidity occurs through RhoG-dependent endocytosis of $\alpha_5\beta_1$ integrin, related to Fig. 5. The effect of syndecan-4 engagement on unbinding energy using fibroblasts transfected with RhoG-targeted siRNA. These experiment are repeats of those displayed in Fig. 5G using an alternative siRNA sequence. Western blots compare RhoG expression between control and siRNA-transfected populations. Values represent at least 10 measurements per condition from 3 separate experiments. Error bars represent standard error.

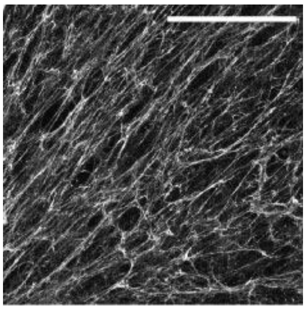

Figure S4. Cell-derived fibrillar matrix, related to Fig. 7. Confluent fibroblasts were cultured for 10 days before removing the cells by NH_4OH lysis. Resultant matrix was stained for fibronectin. Bar = 100 μm.

Supplemental Experimental Procedures

Antibodies and ECM proteins

Mouse monoclonal antibodies raised against pY14-caveolin, clathrin heavy chain, EEA1, Rab4, PKCa (BD Transduction Labs), Arf6, tubulin (DM1A) (Sigma), RhoG (Santa Cruz), HSP70 (Affinity BioReagents), α1 Na^+ K^+ ATPase transporter (Millipore), GFP (Covance) and transferrin receptor (H68.4, Invitrogen; OKT9, European Collection of Cell Cultures), a rat monoclonal antibody against macrophages (F4/80, Abcam) and polyclonal antibodies against caveolin (BD Transduction Labs), dynamin-2, α_5 integrin and RhoGDIα (Santa Cruz) were used according to the manufacturer's instructions. Inhibitory integrin antibodies against human α_5 (mAb16), β_1 (mAb13), and α_v (17E6) were gifts from K.M. Yamada (NIH, USA) and S.L. Goodman (Merck, Darmstadt, Germany). Activating integrin antibodies against human β_1 (9EG7, 12G10 and TS2/16) were from Pharmingen, Abcam and a gift from F. Sanchez-Madrid (Universidad Autonoma de Madrid, Spain), respectively. Non-activity-modulating antibody against β_1 integrin (K20) was from Beckman Coulter. Monoclonal antibody for blotting β_1 integrin (JB1A) was a gift from J.A.Wilkins (University of Manitoba, Canada). Alexa Fluor680-conjugated IgGs were from Molecular Probes (Invitrogen), IR800-conjugated IgGs from Rockland, HRP-conjugated IgGs from DAKO, and biotinylated IgGs from Stratech scientific. As RhoG is a low-abundance protein, when blotting for reduction in RhoG expression by RNAi, it was necessary to immunoprecipitate RhoG from equal volumes of equal-concentration lysate before blotting. Recombinant fibronectin polypeptides encompassing type III repeats 6-10 (50K) (Danen et al., 1995) and 12-15 (H/0) (Sharma et al., 1999) were expressed as recombinant polypeptides as described

previously (Makarem et al., 1994). Bovine plasma fibronectin and heparin were purchased from Sigma.

Cell Culture

Human foreskin fibroblasts, passage number 8-25, were cultured at 37°C in DME, 10% foetal bovine serum, 4.5 g/l glucose, 25 mM HEPES and 2 mM L-glutamine. The generation of immortalised wild-type, *Scd4* -/- and Y188L-syndecan-4-expressing MEFs has been described previously (Bass et al., 2007). To allow expression of the large T antigen, MEFs were cultured at 33°C in DME, 10% foetal bovine serum, 4.5 g/l glucose, 2 mM L-glutamine and 20 U/ml IFNγ (Sigma). Telomerase-immortalised human fibroblasts transfected with GFP-RhoG were cultured at 37°C in DME, 15% foetal bovine serum, 4.5 g/l glucose, 25 mM HEPES and 2 mM L-glutamine.

Primary *Rhog* -/- MEFs were generated by crossing heterozygous parents, isolating E13.5 embryos from knockout, wild-type and heterozygous littermates. Embryos were decapitated and eviscerated. Torsos were macerated and MEFs dissociated by 20-minute digestion in 10 mg/ml trypsin at 37°C. 5 x 10^6 MEFs were seeded into a 25-cm^2 culture dish and cultured in DME, 10% foetal bovine serum, 4.5 g/l glucose, 2 mM L-glutamine and 100 U/ml penicillin/streptomycin for up to 5 passages.

Primary keratinocytes were isolated by removing skins from 1-3 day neonatal mice, and digesting with 2.5 U/ml dispaseII. The epidermis was peeled free, minced and digested with 10 mg/ml trypsin. The dissociated tissue was resuspended in KBM-Gold media (Lonza), passed through a 70-μm nylon filter, and seeded onto collagen IV-coated dishes at 50000 cells/cm^2. Cells were cultured for 2 days, before adding

10% serum to drive keratinocyte differentiation. The confluent monolayer was scratched with a pipette tip for migration analysis.

Matrix-coated surfaces

For atomic force measurement, poly-D-lysine-coated fluorodishes (World Precision Instruments) were coated with patches of 10 μg/ml fibronectin (Sigma) in Dulbecco's PBS containing calcium and magnesium (Sigma) for 1 hour at room temperature, and then patches of 100 μg/ml 50K at 4°C overnight. For biochemical assays and TIRF microscopy, 10-cm tissue culture-treated plastic dishes (Corning BV) or fluorodishes were coated with 10 μg/ml 50K at 4°C overnight. Equivalent coating of fibronectin and 50K onto fluorodishes was tested by ELISA using the anti-fibronectin mAb 333.

Enzyme-linked Immunosorbent Assays (ELISA)

Ligand-coated surfaces were blocked for 30 minutes with 5% BSA in TBS (10 mM Tris-HCl, pH 7.4, 150 mM NaCl, 0.05% NaN_3) before incubating for 1 hour in mAb 333 diluted in blocking solution. Surfaces were rinsed with PBS-, 0.1% Tween-20 before incubating for 30 minutes in anti-Rat HRP diluted in 0.1% BSA in PBS-, and rinsing thoroughly with PBS-, 0.1% Tween-20. The ELISA was developed by addition of 2 mM ABTS, 2.5 mM H_2O_2, 0.1 M NaOAc, 50 mM NaH_2PO_4, pH 5.0, stopped by addition of 1% SDS, and absorbance readings at 405 nm were measured using a multiscan plate reader.

Atomic Force Microscopy

Force measurements were made using a CellHesion200 atomic force head (JPK Instruments) mounted on an Olympus chassis. Cells were mounted on tipless silicon

SPM-sensor cantilevers (Nanoworld) coated with 50K. The spring constant for each cantilever was calculated *in situ* by thermal fluctuation analysis. Cycloheximide-treated cells were resuspended in HAM's F-12 nutrient media, 25 mM HEPES, 25 μg/ml cycloheximide and captured before they could adhere firmly to the dish. Brightfield optics were used to ensure the capture of individual cells, and transfected cells were selected by epifluorescence where appropriate. Captured cells were allowed to rest for 20 minutes before taking measurements and in each case the first measurement discarded. Cantilever-mounted cells were contacted repeatedly with fluorodish coated with patches of fibronectin and 50K, at equivalent ligand density, with an applied force of 1 nN, and a 5-second contact time. Retraction measurements were made over a 90-μm pulling distance with an extension/retraction speed of 5 μm/s. 45-second pauses between measurements allowed the cell to recover. For stimulation experiments, baseline measurements of unbinding from 50K and fibronectin were made before injecting the soluble stimulant into the dish. Stimulants were used at the following final concentrations: H/0, 10 μg/ml; H/0-heparin, 10 μg/ml each; monoclonal antibodies, 10 μg/ml; PMA, 30 μM; MiTMAB, 100 nM (Calbiochem). Force curves were analysed using JPK data analysis software with baseline and tilt correction. For analysis of force required for individual unbinding events, steps were fitted with a significance of $p < 0.001$.

TIRF Microscopy

Internalisation of β_1 integrin-GFP was recorded using a Leica AM TIRF system set at a 70-nm penetration depth with a 100x NA 1.47 lens. Images were acquired at 2 frames per second using a Hamamatsu C9100 EM-CCD camera. The time schedule of image acquisition was: 5 minutes on 50K; addition of H/0 or H/0 complexed with

heparin (10 μg/ml each); image acquisition of the stimulated cells for a further 5 minutes.

Migration Analysis

For migration over cell-derived matrices, cells were seeded at 5000 cells/ml and allowed to spread for 4 hours before capturing time-lapse images at 10-minute intervals for 10 hours on a Leica AS MDW microscope using a 5x NA 0.15 Fluotar objective and Roper CCD camera. For analysis of persistence, the migration paths of all non-dividing, non-clustered cells were tracked using ImageJ software, and persistence was determined by dividing linear displacement of a cell over 10 hours by the total distance migrated.

Western Blotting

Proteins were resolved by SDS-PAGE, transferred to nitrocellulose, and analysed using the Odyssey Western blotting fluorescent detection system (LI-COR Biosciences UK Ltd.). This involved the use of fluorophore-conjugated secondary antibodies that were detected using an infrared imaging system that allowed both an image of the membrane and an accurate count of bound protein to be recorded.

Membrane Fractionation

For each time point, 10^6 fibroblasts were spread on 50K-coated dishes in DME, 25 mM HEPES, 25 μg/ml cycloheximide at 37°C for 2 hours, and then stimulated with 10 μg/ml H/0 for 0-90 minutes. Cells were harvested at 4°C by scraping in Dulbecco's PBS containing calcium and magnesium, cOmplete protease inhibitor (Roche), 5 mM Na_2VO_4, 10 mM NaF. Membranes were fragmented by three 12-J pulses using a

Vibra-Cell sonicator (Sonics), before removing nuclear debris with a 10-minute, 1000 x g centrifugation step. Membranes were separated into plasma membrane pellet and vesicle/soluble supernatant by 10-minute centrifugation at 10000 x g.

GTPase Activation Assays

Active RhoG was affinity precipitated using GST-ELMO2 (amino acids 1-362). For each time point, fibroblasts were spread and stimulated with H/0 as described above before lysis in 50 mM Tris (pH 7.5), 10% (v/v) glycerol, 100 mM NaCl, 2 mM $MgCl_2$, 1% (v/v) Triton X-100, 0.5% (w/v) sodium deoxycholate, 0.1% sodium dodecyl sulfate, cOmplete protease inhibitor. GST-protein-loaded beads were incubated with the lysates for 1 hour and then washed 3 times with lysis buffer. Active, precipitated GTPase was analysed by fluorescent Western blotting.

Integrin Complexes

Multimolecular integrin complexes were isolated using a previously validated procedure (Humphries et al., 2009). 7 x 10^7 4.5-μm tosyl-activated paramagnetic beads (Invitrogen) were coated with 200 μg/ml antibody in 0.1 M sodium phosphate (pH 7.4-8), 0.17% (w/v) BSA at 25°C, 1400 rpm for 24 hours. Free tosyl groups were blocked with 0.2 M Tris-HCl (pH 8.5), 0.1% (w/v) BSA at 25°C, 1400 rpm for 16 hours. Beads were incubated for 20 minutes at room temperature with 3 x 10^7 immortalised fibroblasts expressing GFP-RhoG. Complexes were stabilised for 20 minutes at room temperature with 2.5 mM DTBP membrane-permeable, reducible crosslinker (ThermoFisher). Under these conditions, endocytosis of beads was determined to be <10% by brightfield microscopy. Cells were lysed by sonication in 10 mM PIPES (pH 6.8), 50 mM NaCl, 3 mM $MgCl_2$, 150 mM sucrose, 0.5% (w/v)

Triton X-100, cOmplete protease inhibitor, 5 mM Na_2VO_4, 10 mM NaF. The sonication regime was 3 x 20-second pulses at 40% power (44 J each), and 2 x 30-second pulses at 50% power (145 J each) using a Vibra-Cell sonicator (Sonics). Complete cell disruption was confirmed by brightfield microscopy. Beads were isolated magnetically and washed four times with the lysis buffer, and bound protein analysed by fluorescent Western blotting.

Matrix-regulated integrin complexes were isolated from MEFs expressing β_1 integrin-GFP. 2 x 10^6 cycloheximide-treated MEFs were spread on 50K-coated dishes for 2 hours, before stimulation for 10 minutes with 10 μg/ml H/0 as appropriate. Complexes were stabilised for 15 minutes with 2.5 mM DTBP membrane-permeable, reducible crosslinker. MEFs were lysed for 30 minutes in 50 mM Tris (pH 7.8), 1% (w/v) NP40, cOmplete protease inhibitor and integrin complexes isolated by GFP-Trap for 2 hours (ChromoTek). Beads were washed 3 times with 50 mM Tris (pH 7.8), 50 mM NaCl, 0.7% (w/v) NP40, cOmplete protease inhibitor, and bound protein analysed by fluorescent Western blotting.

Histology

Mice were sacrificed 3 days post-wounding and tissue was fixed in 4% paraformaldehyde for embedding in paraffin. 10-μm sections were subjected to Masson's Trichrome staining or immunohistochemistry using α-smooth muscle actin or F4/80 anti-macrophage antibody, biotinylated secondary antibody, ABC-HRP conjugate and 3,3-diaminobenzidine developing reagent (Vector Laboratories). Cells staining positively for α-SMA were quantified by applying an identical, empirically determined intensity threshold to all images and counting automatically using ImageJ.

RNAi Knockdown

siRNA duplexes with ON TARGET™ modification for enhanced specificity and an siGLO® non-targeting control duplex were purchased from Dharmacon (Thermo Fisher Scientific). Sequences targeted the sense strand of mouse Arf6 (CUGACAUUUGACACGAAUA), PKCa (GAAGGGUUCUCGUAUGUCAUU), caveolin-1 (GCUAUUGGCAAGAUAUUCA), RhoG (GCUUAACCCAACACCGAUA), human dynamin-2 (GAGAUCAGGUGGACACUCU or CCGAAUCAAUCGCAUCUUC), caveolin-1 (GCAUCAACUUGCAGAAAGA or GCAAAUACGUAGACUCGGA), clathrin heavy chain (GCAGAAGAAUCAACGUUAU), and RhoG (CUACACAACUAACGCUUUC or GCUGUGCGCUACCUCGAAU). For knockdown, 80 pmol of targeted oligo and 80 pmol of GLO oligo were transfected into a 90% confluent 25-cm^2 flask using Lipofectamine™2000 reagent (Invitrogen). After 18 hours, the cells were passaged and cultured for 2 days before transfecting again to ensure substantial knockdown. Cells were passaged 18 hours after the second round of transfection and used within 2-3 days. Expression of target proteins in comparison with mock-transfected cells was tested by Western blotting. When capturing cells for atomic force measurements, cells that had taken up the GLO oligo were visualised by epifluorescence.

Supplemental References

Bass, M.D., Roach, K.A., Morgan, M.R., Mostafavi-Pour, Z., Schoen, T., Muramatsu, T., Mayer, U., Ballestrem, C., Spatz, J.P., and Humphries, M.J. (2007). Syndecan-4-dependent Rac1 regulation determines directional migration in response to the extracellular matrix. J Cell Biol *177*, 527-538.
Danen, E.H., Aota, S., van Kraats, A.A., Yamada, K.M., Ruiter, D.J., and van Muijen, G.N. (1995). Requirement for the synergy site for cell adhesion to fibronectin depends on the activation state of integrin alpha 5 beta 1. J Biol Chem *270*, 21612-21618.
Humphries, J.D., Byron, A., Bass, M.D., Craig, S.E., Pinney, J.W., Knight, D., and Humphries, M.J. (2009). Proteomic analysis of integrin-associated complexes identifies RCC2 as a dual regulator of Rac1 and Arf6. Sci Signal *2*, ra51.
Makarem, R., Newham, P., Askari, J.A., Green, L.J., Clements, J., Edwards, M., Humphries, M.J., and Mould, A.P. (1994). Competitive binding of vascular cell adhesion molecule-1 and the HepII/IIICS domain of fibronectin to the integrin alpha 4 beta 1. J Biol Chem *269*, 4005-4011.
Sharma, A., Askari, J.A., Humphries, M.J., Jones, E.Y., and Stuart, D.I. (1999). Crystal structure of a heparin- and integrin-binding segment of human fibronectin. Embo J *18*, 1468-1479.
